# Association between energy-adjusted dietary inflammatory index and increased risk of all-cause Crohn’s disease: a prospective cohort study

**DOI:** 10.3389/fpubh.2026.1792338

**Published:** 2026-04-29

**Authors:** Na Zhao, Fangyuan Hu, Jizhou Liang, Yanrong Zhang, Lei Yuan, Bihan Tang, Jia He, Yuan Liu

**Affiliations:** 1 Department of Ophthalmology, The First Naval Hospital of Southern Theater of PLA, Zhanjiang, China; 2 Faculty of Military Health Service, Naval Medical University, Shanghai, China; 3 Department of Health Statistics, Faculty of Military Health Service, Naval Medical University, Shanghai, China; 4 Department of Gastroenterology, First Affiliated Hospital of Naval Medical University, Shanghai, China; 5 Faculty of Military Health Service, Department of Health Management, Naval Medical University, Shanghai, China

**Keywords:** cox regression models, Crohn’s disease, energy-adjusted inflammatory diet index, prospective cohortstudy, UK Biobank

## Abstract

**Background:**

Crohn’s disease (CD) is a chronic inflammatory bowel condition with increasing global incidence. Diet is a key modulator of chronic inflammation, often assessed by the Dietary Inflammatory Index (DII). However, prospective evidence linking the DII to CD risk remains limited in large populations. This study used the UK Biobank (UKB) to address this gap.

**Methods:**

Cox regression models were used to examine the association between the energy adjusted dietary inflammation index (E-DII) quartiles and CD with sequential adjustment for confounding variables. Restricted cubic spline regression (RCS) was additionally adopted to determine the association of the continuous E-DII and CD risk. Furthermore, sensitivity and subgroup analyses were conducted to explore the consistency of the results.

**Results:**

Among 207,582 participants included in the analysis, 455 incident CD cases were documented over a mean follow-up period of 12.65 years. A positive association was observed between higher E-DII scores, indicating a more pro-inflammatory diet, and an increased risk of CD. In a fully adjusted multivariable model, participants in the highest quartile of E-DII had a significantly elevated risk of CD [HR:1.45, 95% CI (1.11–1.90); *p* < 0.01] compared to those in the lowest quartile. This association remained consistent across several sensitivity analyses.

**Conclusion:**

In this large prospective cohort, a pro-inflammatory dietary pattern, as reflected by higher E-DII scores, was associated with a significantly increased risk of developing CD. From a public health perspective, this finding highlights the potential importance of dietary inflammation in CD prevention, warranting further investigation into targeted interventions.

## 1 Introduction

Crohn’s disease (CD) is classified as a chronic inflammatory bowel disease, characterized by non-specific inflammation in various regions of the gastrointestinal tract. This condition is frequently associated with symptoms including abdominal pain, diarrhea, weight loss, and fatigue, thereby imposing considerable suffering and healthcare burdens on patients (1). Currently, the etiology of CD remains incompletely understood, however, it is widely believed that this complex condition results from the interplay of various factors, including genetic predisposition, environmental influences, and immune system responses (2, 3). In recent years, there has been increasing attention to the role of dietary factors in inflammatory bowel disease (IBD) (3–6). A preliminary analysis indicates that a therapeutic dietary intervention for CD based on Mediterranean dietary principles significantly reduces inflammatory markers and intestinal permeability in patients with mild to moderate active CD, while also altering the composition of gut microbiota (7). A meta-analysis has demonstrated that the symptomatic improvements observed in CD patients following specific dietary interventions are attributed to the anti-inflammatory components. However, the overall quality of evidence remains low, and the effects on inflammatory biomarkers remain inconclusive. Furthermore, existing studies are limited by substantial heterogeneity and inconsistent findings (8). Above evidence suggests that diet may have some influence on CD; however, there is a lack of in-depth research and robust evidence to fully support this relationship.

The Dietary Inflammatory Index (DII) serves as an important tool for assessing the impact of foods on inflammation. By integrating the potential effects of various dietary components on inflammation, it offers a novel perspective for evaluating the contribution of diet to chronic diseases (9, 10). The DII is constructed based on up to 45 dietary parameters (foods and nutrients). Each parameter is assigned a literature-derived inflammatory effect score based on its overall directional effect on six established inflammatory biomarkers: interleukin-1β (IL-1β), IL-4, IL-6, IL-10, tumor necrosis factor-*α* (TNF-α), and C-reactive protein (CRP). A dietary component positively correlated with inflammatory indicators receives a DII score of “+1,” while a negative correlation results in a score of “-1.” If a dietary component has no inflammatory effect, it is assigned a score of “0” (11, 12). A final DII score that is negative indicates anti-inflammatory potential, whereas a positive score suggests pro-inflammatory potential, with higher scores indicating greater inflammatory potential (13). In the application of the Dietary Inflammation Index (DII), a variant known as the Energy-Adjusted Dietary Inflammation Index (E-DII) has emerged. E-DII standardizes the food parameter intake of each participant to a per 1,000 kcal consumption basis, effectively mitigating individual variability in dietary intake resulting from differences in physical activity, body composition, and metabolic efficiency (14).

The association between diet and the risk of CD has become a significant focus of research in recent years. Although previous studies have examined the relationship between dietary factors and CD, systematic investigations into the specific impact of DII on CD remain limited (15, 16). The UK Biobank (UKB), a large-scale prospective cohort study, provides extensive data on diet, lifestyle, and health outcomes (17). Leveraging the UKB cohort, this study aims to investigate the prospective association between the E-DII and the risk of CD, rigorously evaluating this link within a large population sample.

## 2 Materials and methods

### 2.1 Study design and population

UKB is the largest repository of information on genetic and environmental factors related to disease causation or prevention in the United Kingdom. Between 2006 and 2010, the UKB project collected data from 500,000 volunteers aged 40 to 69 across the UK (18). The health and medical records of these participants have been tracked over several decades to investigate the relationships between specific genes, lifestyles, and health conditions.

**Figure 1:**
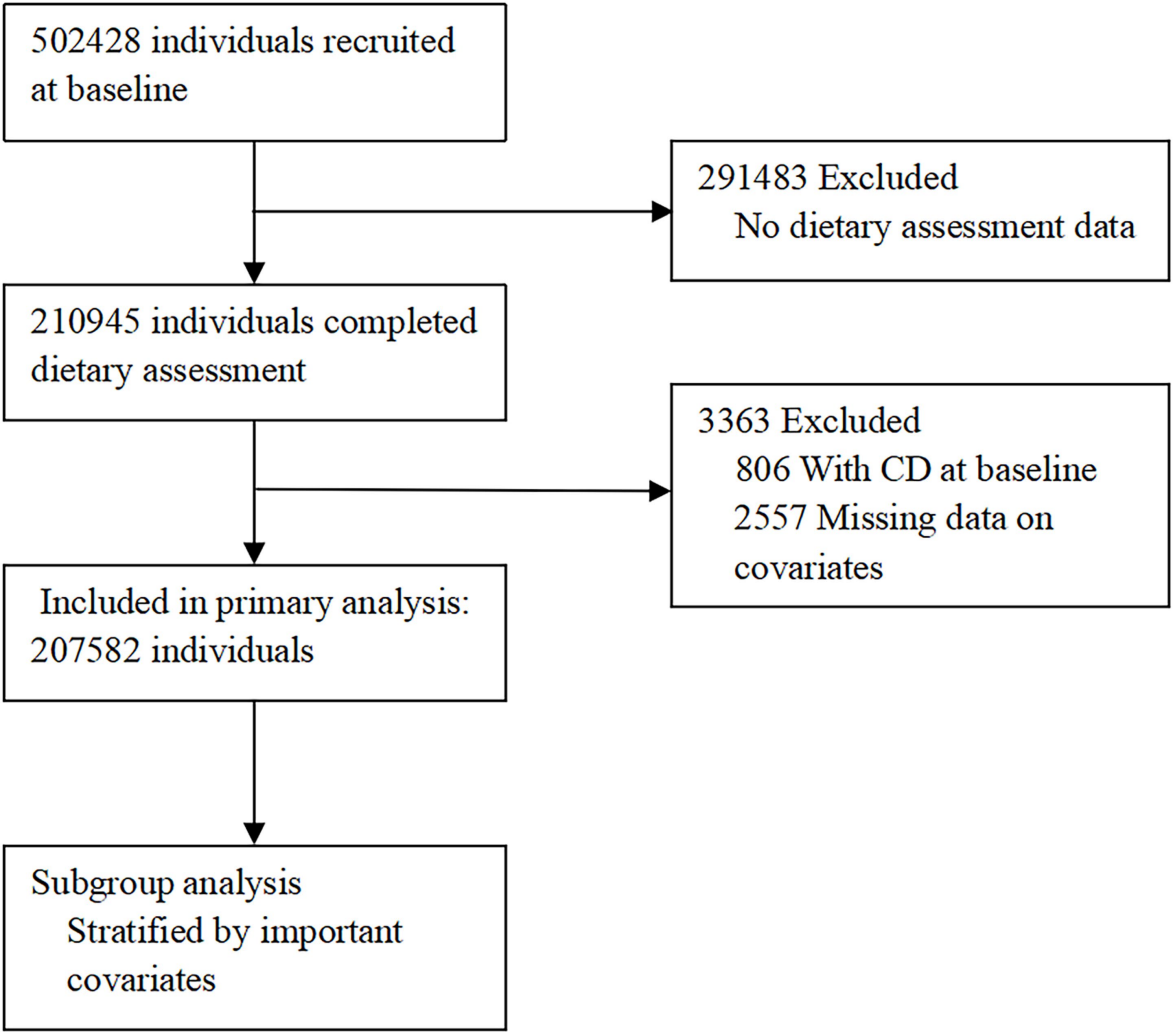
Participant flow chart.

Participants were invited to engage the questionnaire at baseline and on four separate occasions [from the first administration conducted in the assessment centre (April 2009 to September 2010) to the last instance April 2012 to June 2012]. In our study, participants were included if they had completed at least two 24-h dietary recall questionnaires. Exclusion criteria were applied as follows: (1) missing E-DII score records; (2) a diagnosis of CD at baseline, as indicated by self-reported use of medication for CD, a self-reported history of CD, or a CD diagnosis code (ICD-10 K50) recorded in hospital inpatient records or primary care data prior to or at the UK Biobank baseline assessment; or (3) missing data for key baseline covariates. The selection procedure for baseline study participants was depicted in Figure 1. The UKB received approval from the North West Multi-centre Research Ethics Committee. This study was conducted under the UKB resource application number 99709.

### 2.2 Dietary intake assessment

Detailed dietary intake information was obtained from UKB participants to estimate the energy, nutrients, and other food components consumed in the 24 h prior to the interview, using The Oxford WebQs to collect participants’ dietary intake data for up to 206 food items and 32 beverages consumed on the preceding day. To mitigate measurement error bias, the average of all available 24-h recalls (up to a maximum of five) was calculated, and the method of averaging repeated measurements has been previously validated as superior to reliance on a single dietary recall (19).

### 2.3 Calculation of E-DII scores

The DII was designed based on 45 food parameters that have either anti-inflammatory or pro-inflammatory effects, aiming to reflect the dietary inflammatory capacity by combining associations with six inflammatory biomarkers (IL-1β, IL-4, IL-6, IL-10, TNF-*α*, and C-reactive protein) (20). A higher positive DII score indicated stronger pro-inflammatory capacity, while a higher negative score indicated stronger anti-inflammatory capacity. The E-DII scores were calculated by correlating an individual’s average daily intake with the inflammatory effect scores of various food parameters (21, 22). Specifically, the mean daily intake of foods and nutrients derived from WebQs was initially energyc-adjusted using the nutrient density method. Subsequently, each energy-standardized dietary component was weighted by its corresponding inflammatory effect score, as defined by the DII. These weighted values were then aggregated to yield the overall DII and E-DII scores (9, 23, 24). In this study, due to data availability, 26 out of 45 food parameters were selected for the calculation of the DII and E-DII scores. These parameters included alcohol, vitamin B12, vitamin B6, vitamin C, vitamin D, vitamin E, vitamin A, beta carotene, carbohydrate, cholesterol, total fat, fiber, Iron, magnesium, niacin, zinc, omega 3, omega 6, protein, monounsaturated fatty acids, riboflavin, saturated fat, selenium, thiamin, trans fatty acids, and green tea.

### 2.4 Covariates

The covariates for this study were selected based on a review of several published studies (25–28). Demographic data, health status, and lifestyle factors were extracted from the UKB, including variables such as age, gender, BMI, race/ethnicity, educational level, smoking and alcohol consumption history, and significant chronic medical history. Race/ethnicity was categorized as White, African, Asian, Mixed, and Other. Smoking and alcohol consumption were classified based on current, previous, or never use. Data on significant chronic medical history (e.g., hypertension, diabetes, oral diseases, and cancers) were similarly derived from UKB records.

### 2.5 Statistical analysis

Participants were stratified into four groups based on quartiles of their E-DII scores, and the baseline characteristics for these groups were presented as means with standard deviation (SD) for continuous variables and as absolute(n) and relative (%) frequencies for categorical variables. For CD outcomes, participants were followed from baseline until the earliest occurrence of CD diagnosis, loss to follow-up, death, or the end of the study follow-up period (April 2012 to June 2012). Multiple statistical approaches were used to evaluate differences between the non-CD group and the CD group for variables including sex, age at recruitment, BMI, ethnic background, university education, alcohol drinking status, smoking status, diabetes, hypertension, dental problems, and cancer status.

The median and interquartile range (IQR) of E-DII for each nutrient were calculated and presented across the four groups to describe the distribution characteristics of different nutrients. The association between E-DII scores and the risk of CD was estimated using Cox proportional hazards models to obtain hazard ratios (HRs) and 95% confidence intervals (95% CI). We constructed three sequentially adjusted models: Model 1 was adjusted for sex, age at recruitment, ethnic background, and BMI. Building upon Model 1, Model 2 was further adjusted for dental problems, diabetes, hypertension, and cancer. Model 3 additionally included alcohol drinker status, smoking status, and university education. Given the substantial mortality incidence in our cohort, we adopted competing-risks regression methodology to mitigate bias. Using the same methodology, we analyzed the association between DII scores and the risk of CD. To explore potential non-linear relationships, the E-DII scores were also analyzed as a continuous variable using restricted cubic spline (RCS) regression.

Subgroup analyses were conducted using the fully adjusted model (Model 3) to examine heterogeneity across strata defined by sex, age at recruitment, ethnic background, BMI, alcohol drinker status, university education, smoking status, diabetes, hypertension, dental problems, and cancer. Potential non-linear dose–response relationships between E-DII levels and CD risk were further investigated by calculating *p* values for trend.

All analytical and graphical tasks were carried out using SAS 9.4 and R (version 4.2.1). A two-sided *p* value below 0.05 was defined as statistically significant.

## 3 Results

### 3.1 Characteristics of study population in UKB

From the 502,428 participants initially enrolled in the UKB, 291483 were excluded due to lack of dietary assessment data. After further exclusions for missing covariate data (n = 2,557) and baseline CD (n = 806), 207,582 participants were ultimately included in the primary analysis (Figure 1). In the primary analysis, the median of total E-DII in this study was −0.50 with a range of −6.36 to +5.02. Then, the participants were classified into four groups according to the E-DII scores quartile, 51,896 in the lowest-E-DII group (Q1), 51,895 in the lower-E-DII group (Q2), 51,895 in the higher-E-DII group (Q3), 51,896 in the highest-E-DII group (Q4) (Supplementary Table S1). Within the cohort, 455 incident CD cases were documented during follow-up, while the remaining 207,127 individuals served as the non-CD group. The characteristics of the study population were detailed in Table 1. Notably, individuals with a BMI ≥ 30 were more frequently observed in the CD group. Regarding lifestyle factors, never drinkers and current/ previous smokers were both more frequently observed among CD cases, and the prevalence of comorbidities including diabetes, hypertension, cancer, and dental problems was consistently higher in the CD group compared to non-CD group (*p* < 0.05). These observed differences underscored the importance of adjusting for these potential confounders in the multivariable analyses.

**Table 1:** Characteristics of study population in UK Biobank.

Characteristics	Non-Crohn’s disease group (207127) *n* (%)	Crohn’s disease (455) *n* (%)	*p*-value
Sex			0.0917
Male	92,907 (44.86)	222 (48.79)	
Female	114,220 (55.14)	233 (51.21)	
Age at recruitment			0.8015
<60	123,200 (59.48)	268 (58.90)	
≥60	83,927 (40.52)	187 (41.10)	
BMI (kg/m 2)			0.0119
<18.5	1,675 (0.81)	6 (1.32)	
18.5–20	4,346 (2.10)	9 (1.98)	
20–25	71,727 (34.63)	134 (29.45)	
25–30	85,775 (41.41)	183 (40.22)	
≥30	43,604 (21.05)	123 (27.03)	
Ethnic background			0.0694
White	198,496 (95.83)	431 (94.73)	
African	1,018 (0.49)	1 (0.22)	
Asian	3,378 (1.63)	15 (3.30)	
Mixed	1,236 (0.60)	2 (0.44)	
Other ethnic group	2,999 (1.45)	6 (1.32)	
University education			0.0112
No	118,452 (99.76)	88,675 (99.81)	
Yes	287 (0.24)	168 (0.19)	
Alcohol drinker status			0.0005
Current	194,188 (93.75)	407 (89.45)	
Previous	6,289 (3.04)	26 (5.71)	
Never	6,650 (3.21)	22 (4.84)	
Smoking status			0.0018
Current	16,186 (7.81)	48 (10.55)	
Previous	73,622 (35.54)	185 (40.66)	
Never	117,319 (56.64)	222 (48.79)	
Diabetes			<0.0001
No	191,564 (92.49)	397 (87.25)	
Yes	15,563 (7.51)	58 (12.75)	
Hypertension			<0.0001
No	135,290 (65.32)	248 (54.51)	
Yes	71,837 (34.68)	207 (45.49)	
Dental problems			0.0211
No	132,752 (64.09)	268 (58.90)	
Yes	74,375 (35.91)	187 (41.10)	
Cancer			0.0004
No	160,180 (77.33)	320 (70.33)	
Yes	46,947 (22.67)	135 (29.67)	

Data presented as mean (SD, standard deviation) for continuous variables and number (%) for categorical variables. BMI, body mass index.

### 3.2 Nutrients in quartiles of E-DII from study population

Based on E-DII scores, participants were stratified into four investigated groups (Q1–Q4), with Q1 (−6.357–1.700) serving as the reference group, followed by Q2 (−1.700–0.491), Q3 (−0.491–0.610), and Q4 (0.610–5.022). The distribution of each nutrient across these quartiles was shown in Table 2, revealing non-linear relationships. To illustrate, participants in Q1 had a median alcohol intake of 7.10 (IQR = 20.17). These relationships can be categorized into three distinct patterns: (1) alcohol, zinc, omega-6, and protein exhibited an initial increase followed by a decrease with rising E-DII scores; (2) a gradual decreasing trend was seen for vitamin B12, vitamin B6, vitamin C, vitamin D, vitamin E, vitamin A, beta-carotene, fiber, iron, magnesium, niacin, omega-3, riboflavin, selenium, thiamin, and green tea; (3) conversely, carbohydrate, cholesterol, total fat, monounsaturated fatty acids (MUFA), saturated fat, and trans fatty acids manifested a gradually increasing pattern with E-DII scores increasing.

**Table 2:** Nutrients in quartiles of E-DII from study population.

Nutrients	Quartile of E-DII, Median (Quartile)			
	Q1	Q2	Q3	Q4
Alcohol	7.10 (20.17)	9.36 (25.56)	12.15 (30.80)	8.18 (32.04)
Vitamin B12	5.97 (4.03)	5.76 (3.47)	5.59 (3.19)	5.17 (3.07)
Vitamin B6	2.16 (0.80)	2.06 (0.79)	1.97 (0.81)	1.74 (0.86)
Vitamin C	164.26 (98.21)	129.56 (83.45)	102.98 (75.55)	70.99 (67.28)
Vitamin D	3.28 (3.98)	2.99 (3.06)	2.85 (2.65)	2.50 (2.27)
Vitamin E	10.89 (5.29)	10.63 (5.32)	10.38 (5.56)	9.43 (5.94)
Vitamin A	1115.39 (776.54)	756.15 (625.04)	650.13 (499.27)	570.50 (429.98)
Beta carotene	4336.51 (3412.28)	2247.08 (2398.35)	1458.29 (1790.07)	859.68 (1143.01)
Carbohydrate	234.43 (87.96)	247.17 (89.71)	252.24 (95.59)	257.01 (109.13)
Cholesterol	182.30 (145.43)	203.00 (153.15)	218.37 (160.98)	223.18 (173.29)
Total fat	58.99 (28.31)	68.01 (31.01)	74.21 (34.48)	79.79 (40.52)
Fiber	20.74 (8.31)	18.18 (7.22)	16.24 (6.92)	13.71 (6.93)
Iron	12.55 (4.68)	12.32 (4.56)	12.01 (4.61)	11.20 (4.98)
Magnesium	347.57 (115.30)	334.46 (109.14)	319.99 (108.96)	292.47 (115.09)
Niacin	37.73 (13.25)	37.55 (13.31)	37.35 (13.89)	35.29 (15.32)
Zinc	9.33 (3.77)	9.45 (3.84)	9.48 (4.01)	9.15 (4.51)
Omega 3	1.95 (1.33)	1.85 (1.15)	1.80 (1.09)	1.63 (1.07)
Omega 6	9.62 (5.80)	10.34 (5.94)	10.54 (6.09)	9.88 (6.22)
Protein	78.33 (26.73)	79.04 (27.19)	79.56 (28.45)	77.38 (32.38)
MUFA	21.44 (11.18)	24.60 (12.05)	26.73 (13.21)	28.09 (15.23)
Riboflavin	1.89 (0.75)	1.89 (0.74)	1.87 (0.76)	1.76 (0.83)
Saturated fat	19.54 (10.57)	24.20 (12.16)	27.70 (13.96)	32.26 (17.41)
Selenium	51.86 (30.36)	49.29 (27.47)	47.68 (25.85)	43.76 (24.49)
Thiamin	1.93 (0.83)	1.84 (0.80)	1.73 (0.78)	1.52 (0.79)
Trans fatty acids	0.81 (0.62)	1.04 (0.69)	1.18 (0.76)	1.36 (0.93)
Green tea	443.33 (570.00)	418.00 (522.50)	380.00 (601.67)	190.00 (570.00)

Q1, Q2, Q3, Q4 represented four levels of E-DII scores from low to high, Q1 [−6.357--1.700], Q2 [−1.700--0.491], Q3 [−0.491–0.610], Q4 [0.610–5.022].

MUFA, monounsaturated fatty acids.

### 3.3 Associations between E-DII/ DII and CD

Table 3 summarized the association between E-DII quartiles and cumulative incidence of CD, demonstrating statistically significant differences in cumulative CD incidence across quartiles (*p* < 0.01), with participants in higher E-DII quartiles exhibiting progressively increased CD incidence rates. In Model 1, after sex, age at recruitment, ethnic background, and BMI were adjusted, both Cox proportional hazards and Competing Risk models revealed statistically significant differences in CD incidence across E-DII quartile groups (*p* < 0.01). When E-DII was analyzed as a continuous variable, each unit increment in E-DII score was associated with an increased risk of CD [HR:1.11, 95%CI (1.04–1.17), *p* < 0.01] in the Cox model. Nonlinear regression analysis demonstrated a significant association between E-DII and CD risk. (P_Nonlinearity_ < 0.05), suggesting the existence of a nonlinear relationship between E-DII and CD risk. In Model 2, additional adjustments were made for dental problems, diabetes, hypertension, and cancer beyond Model 1 covariates, and both Cox proportional hazards and competing risk models demonstrated persistent significant differences in CD risk across E-DII quartiles (*p* < 0.05). When analyzed continuously, each unit increase in E-DII score remained significantly associated with an elevated CD risk [HR:1.10, 95%CI (1.04–1.17), *p* < 0.01], with nonlinear regression revealing a significant association (P_Nonlinearity_ < 0.05). The inclusion of additional covariates for alcohol drinker status, smoking status and university education in the fully adjusted Model 3 demonstrated that the associations between E-DII quartiles and risk persisted, with statistically significant differences (*p* < 0.05) maintained in both regression models. The continuous E-DII analysis in Model 3 showed a slightly attenuated but still significant positive association [HR:1.09, 95%CI (1.03–1.16), *p* < 0.05], with nonlinear regression confirming the significant relationship (P_Nonlinearity_ < 0.05). Unlike the consistent positive associations observed in E-DII, the original DII did not demonstrate robust associations with CD risk (Supplementary Table S2). Analysis of DII quartiles revealed a significant association only in Model 1, with null associations in Models 2 and 3 after sequential adjustment for potential confounders. Notably, when analyzed as a continuous variable, DII demonstrated a significant positive association with CD risk across all three models, thereby validating the overall findings of this study and reinforcing the robustness of our primary conclusions.

**Table 3:** Associations between E-DII and CD incidence.

Model	Quartile of E-DII, HR (95% CI)				P_Cox-regression_	P_Competing-risks_	Continuous E-DII, HR (95% CI)	P_Cox-regression_	P_Nonlinearity_
	Q1	Q2	Q3	Q4					
Model 1	1 (ref)	1.05(0.79,1.40)	1.23(0.94,1.62)	1.52(1.16,1.98)	0.0008	0.0010	1.11(1.04,1.17)	0.0007	0.0048
Model 2	1 (ref)	1.05(0.79,1.40)	1.23(0.94,1.62)	1.51(1.16,1.97)	0.0009	0.0012	1.10(1.04,1.17)	0.0009	0.0067
Model 3	1 (ref)	1.06(0.80,1.41)	1.23(0.93,1.61)	1.45(1.11,1.90)	0.0028	0.0034	1.09(1.03,1.16)	0.0030	0.0253

Model 1 was adjusted for sex, age at recruitment, ethnic background, BMI.

Model 2 was adjusted for variables in Model 1 plus dental problems, diabetes, hypertension and cancer.

Model 3 was adjusted for variables in Model 2 plus alcohol drinker status, smoking status and university education.

### 3.4 Nonlinear correlation trend between E-DII and CD incidence

**Figure 2:**
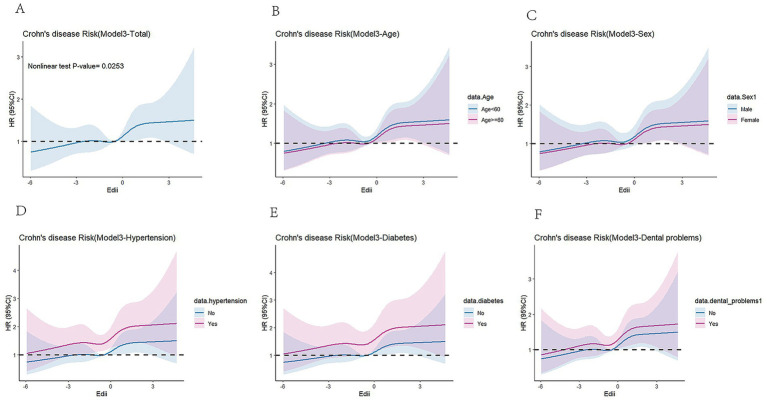
Restricted cubic spline (RCS) model for the hypothesis of nonlinear correlation trend between E-DII and CD incidence. **(A)** Total population, **(B)** population grouped by age, **(C)** population grouped by sex, **(D)** population grouped by hypertension, **(E)** population grouped by diabetes, **(F)** population grouped by dental problems.

When analyzed as a continuous variable with comprehensive adjustment for potential confounders, the E-DII scores demonstrated a statistically significant nonlinear association with CD risk (p_nonlinearity_ < 0.05) (Figure 2). Figure 2A presented the restricted cubic spline analysis from Model 3, illustrating the dose–response relationship between E-DII scores and CD risk. The solid curve represented adjusted hazard ratios, while the shaded area depicted the corresponding 95% CI, revealing a non-linear association that persisted following full adjustment for all covariates in Model 3. Figures 2B–F presented stratified analyses by age, sex, hypertension, diabetes, and dental problems, respectively. Notably, the hazard ratios and 95% CI exhibited consistent patterns across all subgroups—a finding that mirrored the primary analysis in Figure 2A and demonstrated the robustness of our results.

### 3.5 Associations between E-DII and CD incidence in subgroups analysis

**Figure 3:**
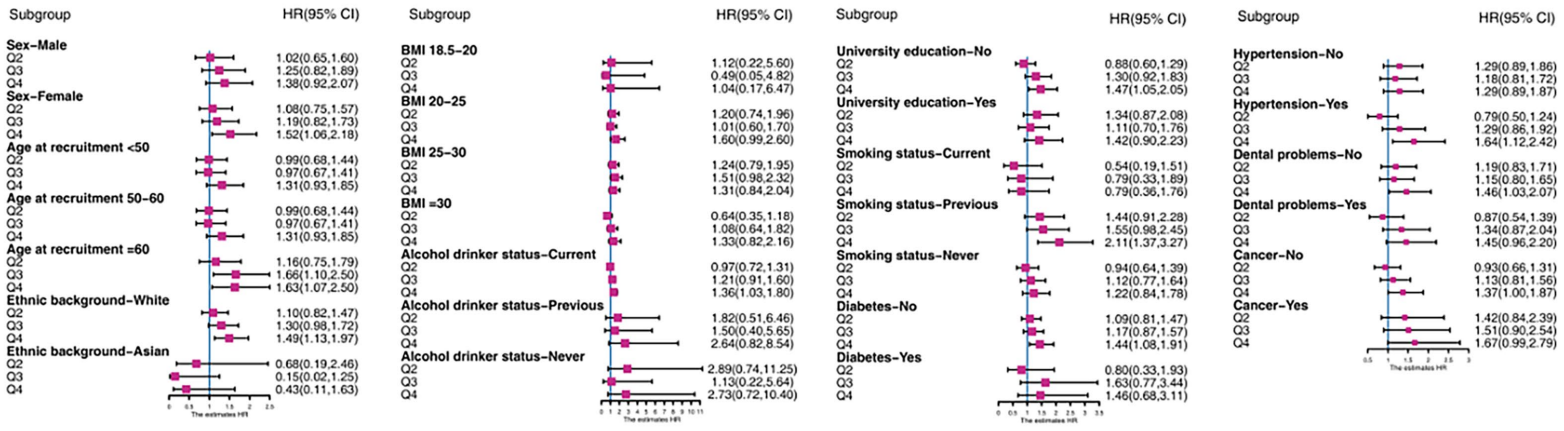
Associations between E-DII and CD incidence in subgroups analysis.

Subgroup analyses for key covariates were undertaken to evaluate the association of E-DII quartiles with CD incidence across these strata, with multivariable adjustment (Figure 3). A robust association was observed, whereby the highest E-DII quartile consistently showed a significantly increased CD risk relative to the lowest quartile (*p* < 0.05 for most comparisons). These subgroup risk estimates were in line with the findings from the primary analysis. Furthermore, a significant dose–response relationship was observed across increasing E-DII quartiles in most subgroups, as evidenced by trend tests yielding p < 0.05 in the majority of analyses.

## 4 Discussion

The UKB is a large-scale, longitudinal prospective cohort comprising comprehensive multidimensional data from over 500,000 participants, featuring long-term follow-up and comprehensive phenotyping data that provide robust statistical power and effectively minimize random errors in statistical analyses. Our analysis established a significant dose–response association between higher E-DII scores and increased CD risk using Cox proportional hazards regression. This finding contributes to a better understanding of CD pathogenesis and provides a scientific basis for considering dietary strategies in disease management. Specifically, a consistent, dose-dependent increase in CD risk was observed with higher E-DII scores. These findings implicate diet-associated inflammation as a key modifiable environmental factor in CD pathogenesis, thereby providing epidemiological support for its environmental etiology. This perspective usefully complements the prior predominant focus on genetic susceptibility and gut microbiota (18, 29).

Previous studies examining the relationship between DII and CD risk have yielded inconsistent findings, with some reporting null associations and others demonstrating significantly increased risk of CD. Using the UKB, Gu et al. (30) reported a higher E-DII score was not associated with risk of CD or ulcerative colitis (UC) in the overall population, observing an association only among participants with high genetic susceptibility. Similarly, Wellens et al. (31) found null associations between empirical dietary inflammatory pattern (EDIP) and DII and IBD onset or IBD-related outcomes in the large prospective UK cohort. In contrast, Lo et al. (15) utilizing the EDIP scores in three prospective cohorts from the United States, reported dietary patterns with high inflammatory potential to be associated with increased risk of CD but not UC. More recently, a systematic review and meta-analysis by Meyer et al. (32) synthesized evidence from observational studies and confirmed that inflammatory diet and ultra-processed foods were associated with an increased risk of CD. These discrepancies may be attributable to differences in index construction—particularly whether energy adjustment was applied—as well as variations in dietary assessment methods. In our study, the E-DII, which accounted for total energy intake, demonstrated a robust positive association with CD risk across all models, whereas the original DII showed significant associations only when analyzed as a continuous variable, with quartile-based analyses yielding null findings after full covariate adjustment. This pattern suggested that energy adjustment may enhance the precision of dietary inflammatory potential estimates by reducing confounding due to variations in overall energy intake, which may explain the stronger and more consistent associations observed for E-DII in our study. Notably, the inconsistencies between our findings and those of previous studies may be attributable to several methodological factors. Gu et al. (30), despite using the E-DII score, reported null associations with CD risk, which may be explained by their relatively smaller sample size and inadequate adjustment for potential confounders. Lo et al. (15), utilizing the EDIP score in three prospective US cohorts, found that pro-inflammatory dietary patterns were associated with an increased risk of CD. The divergence between their results and ours may stem from differences in study populations: their cohorts consisted predominantly of health professionals (e.g., nurses), a relatively homogeneous group that may not fully reflect the general population. In contrast, our study employed the E-DII in a larger and more diverse population-based UKB cohort, which likely provides greater generalizability and more reliable estimates.

Furthermore, RCS analysis revealed a nonlinear dose–response relationship between E-DII and CD risk, with trends consistent with the Cox model predictions. Collectively, above findings suggest that pro-inflammatory diets may serve as an independent risk factor for CD onset, even after adjustment for known confounders (33). To further validate this association, longitudinal dietary intervention studies are warranted to assess whether anti-inflammatory dietary modifications can effectively reduce CD incidence in high-risk populations.

Subgroup analyses revealed significant heterogeneity in the association between dietary inflammatory potential and CD risk across populations with different comorbidities. Notably, individuals with pre-existing conditions exhibited markedly higher CD risk at equivalent E-DII scores compared to their healthy counterparts. The discrepancy in dental problems observed between the two groups may be attributable to the following two factors: (1) Pathological alterations in the oral bacterial microbiota, such as those occurring during periodontal disease, are implicated in elevated oral inflammation, promoting the progression of inflammatory bowel disease. (2) Bacteria associated with oral diseases can be translocated to the gut, where they may directly exacerbate inflammatory bowel disease (34).

Several limitations of this study should be acknowledged. As an observational analysis, the causal relationship between dietary factors and CD risk cannot be definitively established. Large-scale longitudinal studies and randomized controlled trials are required to identify optimal dietary strategies for different patient subgroups and to investigate their long-term effects on disease progression (35). Although the UKB comprehensively collected baseline participant information, certain potential confounding factors (e.g., history of intestinal parasite infections, chronic stress levels) may not have been adequately documented. These unmeasured confounders could potentially bias the Cox model’s estimates of true associations by altering dietary patterns or inflammatory status (36). Future research should incorporate additional cohort data or employ alternative approaches such as Mendelian randomization to further validate the robustness of these associations.

Collectively, this study provides novel insights into the role of E-DII in CD, highlighting the potential of dietary modification as a critical component of comprehensive disease management. Further investigation into the underlying molecular mechanisms and development of personalized dietary regimens will lay the groundwork for more effective preventive and therapeutic strategies against CD.

## 5 Conclusion

In summary, our study demonstrates that pro-inflammatory diets, as quantified by the E-DII, are independently associated with an increased risk of CD, even after comprehensive adjustment for potential confounders. These findings underscore the importance of dietary modification as a modifiable factor in CD prevention and management. Future research should focus on elucidating the underlying molecular mechanisms and evaluating the efficacy of personalized anti-inflammatory dietary interventions in high-risk populations. Ultimately, integrating nutritional strategies with conventional therapies may pave the way for more effective, holistic approaches to CD prevention and care.

## Data Availability

For UKB data, public sharing is restricted. Access to these confidential data is governed by the UK Biobank Institutional Data Access/Ethics Committee (http://www.ukbiobank.ac.uk).
