# Supplementary material for: Association between energy-adjusted dietary inflammatory index and increased risk of all-cause Crohn’s disease: a prospective cohort study
Source: Front Public Health. 2026 Apr 29;14:1792338. doi: 10.3389/fpubh.2026.1792338 (PMC13167928; doi:10.3389/fpubh.2026.1792338)
Supplement: Supplementary file 1 [file Supplementary_file_1.pdf]

Association between energy-adjusted dietary inflammatory index and increased risk of all-cause crohn's disease: a prospective cohort study

E-DII:
Q1[-6.357--1.700]
Q2[-1.700--0.491]
Q3[-0.491-0.610]
Q4[0.610- 5.022]

Table S1. Characteristics of study population in UK Biobank

Characteristics	Q1 (51896) n (%)	Q2 (51895) n (%)	Q3 (51895) n (%)	Q4 (51896) n (%)
**Sex**				
Male	17201 (33.15)	21962 (42.32)	25640 (49.41)	28326 (54.58)
Female	34695 (66.85)	29933 (57.68)	26255 (50.59)	23570 (45.42)
**Age at recruitment**				
<60	27647 (53.27)	29565 (56.97)	31672 (61.03)	34584 (66.64)
⩾60	24249 (46.73)	22330 (43.03)	20223 (38.97)	17312 (33.36)
**BMI(kg/m 2)**				
<18.5	480(0.92)	401(0.77)	376(0.72)	424(0.82)
18.5-20	1325(2.55)	1154(2.22)	971(1.87)	905(1.74)
20-25	19907(38.36)	18987(36.59)	17635(33.98)	15332(29.54)
25-30	20588(39.67)	21464(41.36)	21823(42.05)	22083(42.55)
⩾30	9596(18.49)	9889(19.06)	11090(21.37)	13152(25.34)
**Ethnic background**				
White	49745 (95.86)	50081 (96.50)	49857 (96.07)	49244 (94.89)
African	293 (0.56)	213 (0.41)	226 (0.44)	287 (0.55)
Asian	812 (1.56)	770 (1.48)	828 (1.60)	983 (1.89)
Mixed	284 (0.55)	259 (0.50)	302 (0.58)	393 (0.76)
Other ethnic group	762 (1.47)	572 (1.10)	682 (1.31)	989 (1.91)
**University education**				
No	28983 (55.85)	28209 (54.36)	29213 (56.29)	32334 (62.31)
Yes	22913 (44.15)	23686 (45.64)	22682 (43.71)	19562 (37.69)
**Alcohol drinker status**				
Current	48424 (93.31)	49021 (94.46)	49042 (94.50)	48108 (92.70)
Previous	1632 (3.14)	1404 (2.71)	1365 (2.63)	1914 (3.69)
Never	1840 (3.55)	1470 (2.83)	1488 (2.87)	1874 (3.61)
**Smoking status**				
Current	2665 (5.14)	3113 (6.00)	3956 (7.62)	6500 (12.53)
Previous	18725 (36.08)	18593 (35.83)	18598 (35.84)	17891 (34.47)

Never	30506 (58.78)	30189 (58.17)	29341 (56.54)	27505 (53.00)
**Diabetes**				
No	48248 (92.97)	48185 (92.85)	47983 (92.46)	47545 (91.62)
Yes	3648 (7.03)	3710 (7.15)	3912 (7.54)	4351 (8.38)
**Hypertension**				
No	33711 (64.96)	34105 (65.72)	34006 (65.53)	33716 (64.97)
Yes	18185 (35.04)	17790 (34.28)	17889 (34.47)	18180 (35.03)
**Dental problems**				
No	34463 (66.41)	33802 (65.14)	33154 (63.89)	31601 (60.89)
Yes	17433 (33.59)	18093 (34.86)	18741 (36.11)	20295 (39.11)
**Cancer**				
No	39623 (76.35)	39858 (76.81)	40309 (77.67)	40710 (78.45)
Yes	12273 (23.65)	12037 (23.19)	11586 (22.33)	11186 (21.55)

Table S2. Associations between DII and Crohn's disease incidence

**Model**	**Quintile of DII, HR (95% CI)**				$P_{Cox\text{-}regression}$	$P_{Competing\text{-}Risks}$	**Continuous DII, HR (95% CI)**	$P_{Cox\text{-}regression}$	$P_{Nonlinearity}$
	Q1	Q2	Q3	Q4					
Model 1	1 (ref)	0.73(0.55,0.96)	0.95(0.73,1.23)	1.21(0.94,1.54)	0.0425	0.0620	1.093(1.029,1.162)	0.0042	0.0467
Model 2	1 (ref)	0.73(0.55,0.96)	0.94(0.73,1.22)	1.19(0.93,1.52)	0.0549	0.0780	1.088(1.024,1.156)	0.0068	0.0043
Model 3	1 (ref)	0.72(0.54,0.95)	0.92(0.71,1.20)	1.12(0.87,1.44)	0.1444	0.1800	1.069(1.005,1.137)	0.0337	0.0515

Model 1: Sex, Age at recruitment, Ethnic background, BMI.

Model 2: Variables in Model 1 plus Dental problems, Diabetes, Hypertension and Cancer.

Model 3: Variables in Model 2 plus Alcohol drinker status, Smoking status and University education.
